# Application of mixsep software package: Performance verification of male-mixed DNA analysis

**DOI:** 10.3892/mmr.2015.3710

**Published:** 2015-04-30

**Authors:** NA HU, BIN CONG, TAO GAO, YU CHEN, JUNYI SHEN, SHUJIN LI, CHUNLING MA

**Affiliations:** 1 Department of Forensic Medicine, Hebei Key Laboratory of Forensic Medicine, Hebei Medical University, Shijiazhuang, Hebei 050017, P.R. China; 2 Department of Statistics, Institute of Statistics, Renmin University of China, Beijing 100872, P.R. China; 3 Laboratory of Forensic DNA Testing, Institute of Forensic Science, Public Security Department of Shanxi, Taiyuan, Shanxi 030001, P.R. China

**Keywords:** forensic genetics, short tandem repeats, male-mixed DNA, R package, mixsep package, average peak height/area of the active alleles, mixture proportion, allelic drop-out

## Abstract

An experimental model of male-mixed DNA (n=297) was constructed according to the mixed DNA construction principle. This comprised the use of the Applied Biosystems (ABI) 7500 quantitative polymerase chain reaction system, with scientific validation of mixture proportion (Mx; root-mean-square error ≤0.02). Statistical analysis was performed on locus separation accuracy using mixsep, a DNA mixture separation R-package, and the analytical performance of mixsep was assessed by examining the data distribution pattern of different mixed gradients, short tandem repeat (STR) loci and mixed DNA types. The results showed that locus separation accuracy had a negative linear correlation with the mixed gradient (R^2^=−0.7121). With increasing mixed gradient imbalance, locus separation accuracy first increased and then decreased, with the highest value detected at a gradient of 1:3 (≥90%). The mixed gradient, which is the theoretical Mx, was one of the primary factors that influenced the success of mixed DNA analysis. Among the 16 STR loci detected by Identifiler^®^, the separation accuracy was relatively high (>88%) for loci D5S818, D8S1179 and FGA, whereas the median separation accuracy value was lowest for the D7S820 locus. STR loci with relatively large numbers of allelic drop-out (ADO; >15) were all located in the yellow and red channels, including loci D18S51, D19S433, FGA, TPOX and vWA. These five loci featured low allele peak heights, which was consistent with the low sensitivity of the ABI 3130xl Genetic Analyzer to yellow and red fluorescence. The locus separation accuracy of the mixsep package was substantially different with and without the inclusion of ADO loci; inclusion of ADO significantly reduced the analytical performance of the mixsep package, which was consistent with the lack of an ADO functional module in this software. The present study demonstrated that the mixsep software had a number of advantages and was recommended for analysis of mixed DNA. This software was easy to operate and produced understandable results with a degree of controllability.

## Introduction

The R programming language, which was created as a branch of the S language in the 1980s, is widely used in the field of statistics. R is a free open source software environment that is part of the Gnu’s Not Unix project. As an implementation of the S programming language, R has a complete software system for data processing, statistical computing and graphics functions (1). The primary functions of R include data storage and processing systems. It also has an array of operation tools (among which vector and matrix operations are particularly powerful functions), statistical analysis tools, statistical graphics functions and a simple powerful programming language function, which can control data input and output in order to achieve branch and cycling.

The source code for R is freely downloadable and compiled executable files are available online. R is available for multiple computer platforms, including UNIX (FreeBSD and Linux), Windows and MacOS. R predominantly runs through commands and a number of versions of the graphical user interface have been developed, among which Rstudio is the most commonly used (http://www.rstudio.com) (2). In addition, the Comprehensive R Archive Network (CRAN; http://cran.r-project.org) provides a collection of downloadable executable file version source codes and documentations for R, as well as various software packages written by R users. There are >100 CRAN mirrors worldwide, which are responsible for shunting the primary R server. There are five CRAN mirrors in China, allowing Chinese users to quickly download the R-package.

In bioinformatics, the R language is commonly used for the analysis of molecular biological data. The Bioconductor project (3), which uses R as a genome analysis tool, has been available since its launch in 2001 and is updated twice per year (http://www.bioconductor.org). At present, the Bioconductor project is used in bioinformatics analysis of high-throughput data, microarray data and sequential data, with a large number of metadata packages for pathways, microarrays, genetic markers and organs (3–11). The purpose of the Bioconductor project is to provide powerful statistical analysis and graphics functions for genomic data analysis in order to efficiently analyze metadata in various species and to provide a common platform for bioinformatics.

**Figure 1:**
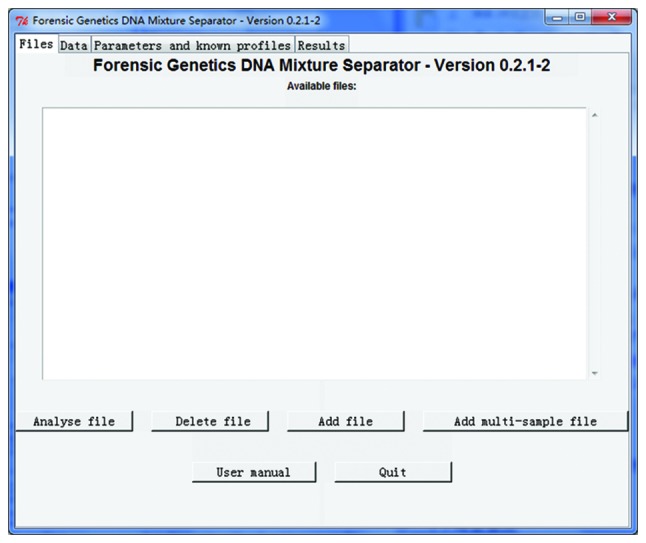
User interface of mixsep package (full name: Forensic Genetics DNA Mixture Separator).

The mixsep package (12–15) is a DNA mixture separator in R, which is developed and maintained by Dr Torben Tvedebrink (Aalborg University, Aalborg, Denmark). This software is a forensic genetics tool used for the analysis of mixed DNA. The present study used the mixsep version 0.2.1-2, updated on May 3, 2013. The user interface of the present version is shown in Fig. 1; URL, http://cran.r-project.org/web/packages/mixsep/index.html; reference manual, http://cran.r-project.org/web/packages/mixsep/mixsep.pdf (12). The mixsep package constructs a statistical model of a greedy algorithm (13) that separates and infers the majority of two-person mixed DNA profiles (separation results are often not unique) on the premise that it does not consider the influence of allelic drop-out (ADO; the low level of a specific DNA content, which may cause relative fluorescence that is too low and may not be separated from the background, therefore providing results in the loss of allelic peak, expressing a false homozygote), stutter and drop-in (DNA contamination), and then conducts the individual identification of mixed DNA. The mixsep package also includes a module for use in complex mixed DNA analysis (more than three people), which has shown limited analytical performance in experimental data validation.

## Materials and methods

### DNA sample collection

Anti-coagulated blood samples (5 ml) were collected from 40 unrelated healthy males at the Blood Center of Hebei Province (Shijiazhuang, China).

### Experimental design

DNA was extracted from each of the 40 whole blood samples and quantified using the ABI 7500 quantitative polymerase chain reaction (qPCR) system (Life Technologies Inc., Carlsbad, CA, USA). Single DNA samples were classified according to whether there were minimal differences in DNA concentrations (<0.5 ng/*µ*l) and then used to generate simulated male-mixed DNA samples of two individuals. This approach allowed the preparation of different mixed DNA gradients by adjusting the volume of DNA solution. To avoid potential over-fitting in statistical analysis caused by single sample type and inadequate sample size, various combinations of mixed DNA samples were generated using different sources (individuals), and each of these combinations was prepared in multiple mixed gradients. This procedure ensured that the influence of mixed DNA profiles and mixed gradients was objectively reflected in the analytical performance of the mixsep software. In addition, the concentration of simulated mixed DNA stock solutions was adjusted to desired levels within the range of 0.5–1.25 ng/*µ*l (that is, the working solution concentration), to achieve the DNA template quantity required by the DNA testing kits.

### Establishment of male-mixed DNA model

#### DNA extraction

DNA was extracted from 40 whole blood samples using an Invitrogen^®^ PureLink™ Genomic DNA Mini kit (Life Technologies Inc.). Aliquots (20 *µ*l) of the 40 DNA extracts (nos. 1-40) were diluted by adding nine volumes (180 *µ*l) of Ambion^®^ Nuclease-Free Water (Life Technologies Inc.) to obtain 10-fold dilutions of the DNA solutions (final volume, 200 *µ*l). The Promega^®^ stock solutions 9948 Male DNA and 2800M control DNA standards (10 ng/*µ*l; 25 *µ*l; Promega, Corp., Madison, WI, USA) were added with nine volumes (225 *µ*l) of Ambion^®^ Nuclease-Free Water, to obtain 10-fold dilutions of the standard samples (final volume, 250 *µ*l).

#### DNA quantification

DNA quantification was performed using the Quantifiler^®^ Human DNA Quantification kit (Life Technologies Inc.), containing DNA standard (200 ng/*µ*l), Human Primer mix, and PCR Reaction mix. Human Primer mix (10.5 *µ*l/sample) and PCR Reaction mix (12.5 *µ*l/sample) were mixed and dispensed into reaction wells (23 *µ*l) followed by the addition of 2 *µ*l sample or standard to each well, in order to obtain a 25-*µ*l PCR reaction mixture. DNA quantification was repeated three times for each sample, and the mean of these was taken as the final DNA concentration.

#### Principles of mixed DNA preparation

Simulated male-mixed DNA was prepared by classifying DNA quantification results of the 40 male samples (nos. 1-40) and the Promega Male-DNA standard; the classification criterion was that single DNA samples have similar concentrations (difference, ≤0.5 ng/*µ*l). The prepared, simulated male-mixed DNA was quantified by ABI 7500 real-time PCR system (Applied Biosystems). The concentration of DNA templates was adjusted to 0.5–1.25 ng/*µ*l as recommended in the instructions for the AmpFlSTR^®^ Identifiler^®^ PCR Amplification kit and the simulated mixed DNA was further diluted whenever necessary.

#### Identifiler PCR and electrophoresis

The 25-*µ*l PCR system contained 10.5 *µ*l PCR Reaction mix, 5.5 *µ*l Identifiler Primer set, 0.5 *µ*l Gold^®^ DNA Polymerase, 9.0 *µ*l Nuclease-Free Water and 1 *µ*l template DNA. Identifiler PCR amplification was performed according to the following conditions: Pre-denaturation at 95°C for 11 min, 28 cycles of denaturation at 94°C for 1 min, annealing at 59°C for 1 min, extension at 72°C for 1 min and a final extension step at 60°C for 60 min. The AmpFlSTR^®^ Identifiler (Life Technologies) PCR products were checked using a 10-*µ*l electrophoresis system containing 0.25 *µ*l GeneScan™, 500 LIZ^®^ Size Standard, 9.25 *µ*l Hi-Di™ formamide and 0.50 *µ*l of PCR product or Allelic Ladder. Capillary electrophoresis was performed on an ABI 3130xl Genetic Analyzer (Applied Biosystems Life Technologies, Foster City, CA, USA). All PCR reagents were purchased from Invitrogen Life Technologies Inc. (Carlsbad, CA, USA).

### Software operation of mixsep

#### Rationale for use

According to the required significance level for statistical analysis, the mixsep package provided the optimal and alternative genotype combinations of short tandem repeat (STR) loci, estimated the parameter of mixture proportion (Mx), fitted the residual peak area error and calculated goodness of fit. Additionally, the mixsep package screened out and removed STR loci with poor goodness of fit, which contributed to the overall variance.

#### Package downloading and installation

The mixsep package for windows was obtained at http://cran.r-project.org/bin/windows/base/release.htm. Installation was accomplished by following the instructions or running the command ‘install.packages’ (‘mixsep’, repo = ‘http://mirrors.ustc.edu.cn/CRAN/’). Mixsep was loaded by running the command ‘library (mixsep)’.

#### Data formatting and loading

**Figure 2:**
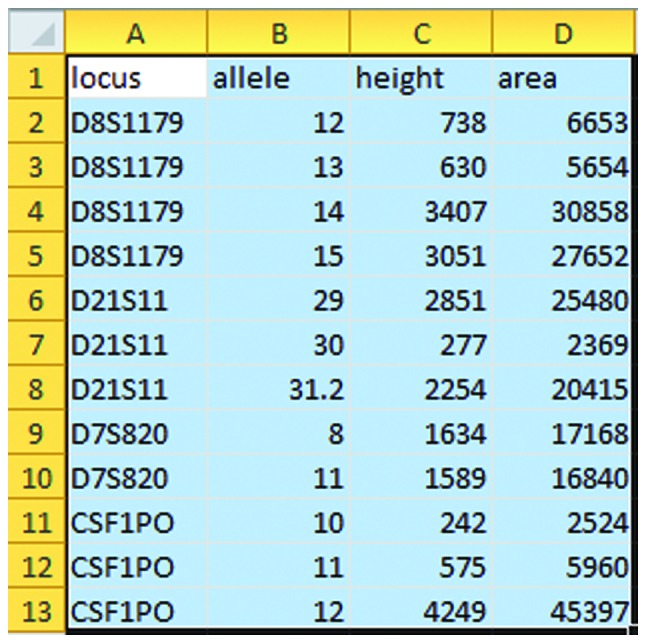
Experimental data saved in the format of a csv file, including four variables: Locus, allele, height and area.

Experimental data were saved as a CSV file containing six variables. These were: Locus, allele, height, area, bp and dye. In the majority of cases, data analysis was performed using the first four of these, as shown in Fig. 2. Data were loaded as a CSV file by clicking ‘Add file’.

#### Variables and genetic marker selection

The variables of locus and allele were required, height and area were alternative, and bp and dye were optional. A DNA testing kit (such as the Identifiler PCR Amplification kit) was selected prior to clicking ‘select column (and kit)’.

#### Selecting loci and alleles

The mixsep default setting analyzed all loci and alleles. Specific loci and alleles were selected whenever necessary and the parameter setting interface was entered by clicking ‘continue’.

#### Parameter setting and mixed DNA analysis

These included ‘Number of contributors’, ‘Search for alternatives’, ‘Specify significance level’, and ‘Use fixed profile’. Mixed DNA analysis was started by clicking ‘Analyze mixture!’.

### Parameters of analytical performance for mixsep

#### Rationale for use

The primary function of mixsep, which lacks a function module for ADO, is the separation of mixed DNA genotype combinations. Therefore, the simulated mixed DNA profiles of STR loci (n = 4566) were statistically analyzed excluding ADO.

#### Locus separation accuracy

Locus separation accuracy refers to an accurate separation of the genotype combination for a specific locus in a sample of mixed DNA profiles.

#### Horizontal analysis

The mixed DNA profile was used as a unit for statistical analysis of locus separation accuracy in order to compare the distribution patterns of the DNA profile data in association with different mixed gradients and mixed sample types.

#### Vertical analysis

The STR locus was used as a unit for the statistical analysis of locus separation accuracy in order to compare the distribution patterns of DNA profile data in association with the 16 STR loci used in the present study.

The separation efficiency of mixsep in male-mixed DNA profiles was assessed using statistical analysis in the horizontal and vertical dimensions.

## Results

### Preparation of simulated male-mixed DNA

The male DNA samples (n=40; nos. 1-40) and Promega male-DNA standards were classified according to the criterion of a DNA concentration difference of no greater than 0.5 ng/*µ*l. The 22 single DNA samples that met this criterion were prepared into eleven groups of two-male mixed DNA samples. To include the Promega male-DNA standard in constructing simulated mixed DNA, the ten-fold-diluted 2800M control DNA working solution was further diluted twice, yielding a final concentration of 0.243 ng/*µ*l (Table I). Each group of male-mixed DNA was prepared into nine mixed gradients, and the samples of each mixed gradient were amplified by PCR three times (thus, n=297). The mixed gradients of male-mixed DNA samples are shown in Table II.

### DNA quantity of male-mixed DNA

The simulated male-mixed DNA samples were checked by assessing selected samples using an ABI 7500 qPCR system (Applied Biosystems), including eleven groups of male-mixed DNA at a mixed gradient of 1:9. DNA quantification of each sample was repeated three times, and the mean values were taken as the DNA concentration (Table III).

To fit the concentration range (0.5–1.25 ng/*µ*l) of template DNA recommended by the kit used in this study, 99 male-mixed DNA working solutions (eleven groups of mixed DNA with nine mixed gradients in each group) were diluted appropriately. According to the DNA quantification results (Table III), 2-*µ*l aliquots of each mixed DNA working solution were diluted by 10- or 15-fold with 9 or 14 volumes (18 or 28 *µ*l) Ambion Nuclease-free Water. The 9948 and 2800M DNA standards with concentrations >0.5 ng/*µ*l were not diluted. The volume of the DNA template was 2 *µ*l for the mixed DNA sample, Sample 11, which was composed of the male-DNA standards (n=27), and 1 *µ*l for the other groups, including single DNA samples used for constructing male-mixed DNA.

### Scientific validation of simulated mixed DNA model

Mx assessment compares the estimated Mx value of the mixsep package (the alpha value) with the pre-set mixed gradient of simulated mixed DNA (the theoretical Mx value) for scientific validation of the established experimental model.

In the present study, the estimated Mx values of mixsep were used as the estimated alpha and the pre-set mixed gradients of male-mixed DNA were used as the theoretical alpha. The distribution of estimated and theoretical alpha values in Identifiler (ID)-STR profiles of the mixed DNA was examined by excluding STR loci with ADO.

**Figure 3:**
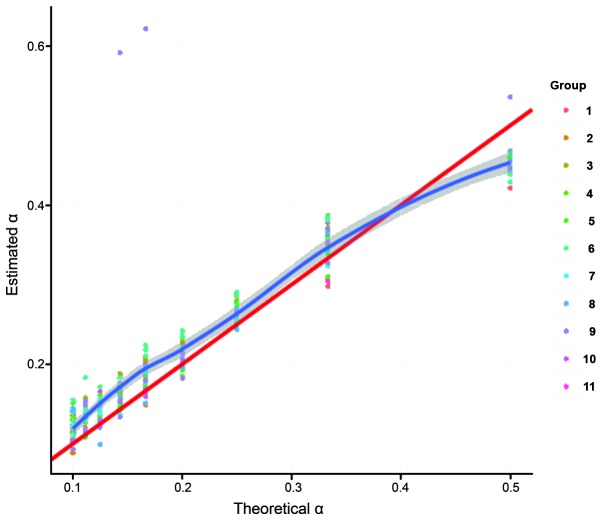
Correlation between estimated and theoretical alpha in male-mixed DNA profiles. The red line indicates y=x, and the blue line represents the locally weighed regression curve.

In Fig. 3, the red line indicates y=x and the blue line represents the locally weighted regression curve. This approach had acceptable anti-noise performance and thus accurately reflected the correlation between estimated and theoretical alpha values. The results showed that with a theoretical alpha value ≤0.33 (that is, mixed gradients of 1:2 to 1:9), the estimated alpha of mixsep was greater than that of the theoretical value. However, with a gradient of 1:1, the estimated alpha value was smaller than that of the theoretical value. This observation may have been based on the assumption of normal distribution in constructing statistical models by mixsep, which led to conservative estimation of relatively extreme mixture proportions (such as 1:5, 1:6, 1:7, 1:8 and 1:9), inclining toward relatively balanced mixture proportions.

Two values showed an abnormal distribution in Fig. 3 and significantly deviated from the locally weighted regression curve. These two data corresponded to the third repetition of the gradient of 1:5 and the first repetition of the gradient of 1:6 for the mixed DNA samples of group no. 9, respectively. The two abnormal data were obtained when running mixsep with source code. However, when running mixsep from the software interface, the obtained alpha values were 0.1742 and 0.1537, respectively, which were each located near the weighted regression curve and followed a normal distribution. The reason for this result is elusive, since all other alpha values estimated using mixsep through source code were consistent with those estimated when using it through the software interface, and no bug was found when running mixsep through the software interface. In view of this situation, the results estimated by mixsep through the software interface are referred to in this article.

Root mean square error (RMSE) statistics showed that in ID-STR profiles, large RMSEs of estimated alpha values are scattered in eleven groups of male-mixed DNA samples, with relatively high frequencies in groups 8 and 9. In terms of mixed gradients, RMSEs were relatively large at a mixed gradient of 1:1 (>0.02) and ranged from 0.01 to 0.02 at the other gradients. Theoretically, mixed DNA at a gradient of 1:1 cannot be accurately separated (although this is ignored in statistical analysis). These results demonstrated that the RMSE between estimated and theoretical Mx was small (≤0.02) in ID-STR profiles of the male-mixed DNA model established in the present study. Thus, the obtained ID-STR profile data did allow scientific and rational analysis of mixed DNA.

### Performance analysis of mixsep

#### Horizontal analysis

**Figure 4:**
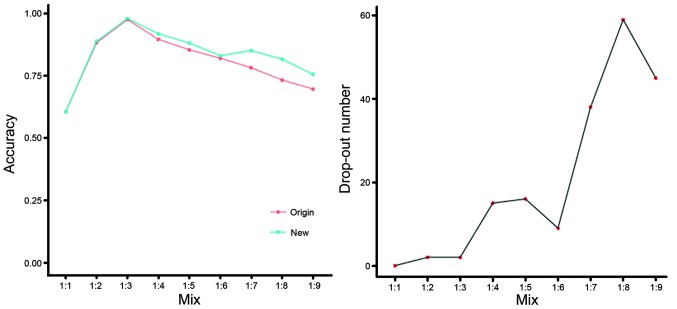
Data distribution of locus separation accuracy (left) and allele drop-out number (right) in male-mixed DNA with nine mixed gradients. The green line represents the distribution pattern excluding the STR loci with drop-out and the red line represents the pattern including the STR loci with drop-out. STR, short tandem repeats.

**Figure 5:**
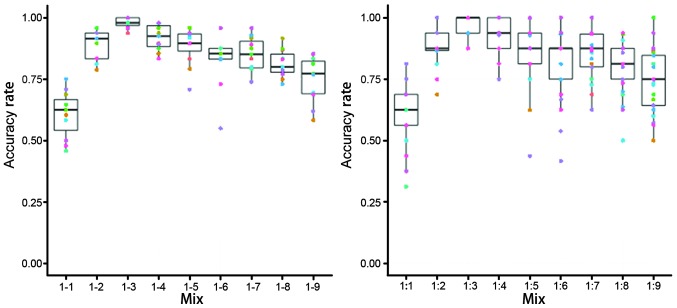
Box-whisker plots of average locus separation accuracy in male-mixed DNA with nine mixed gradients (left) and the results of the three parallel tests in each gradients (right).

The eleven groups of male-mixed DNA profiles (with three parallel tests) at each mixed gradient involved 528 STR loci. Data statistics (Table VI) and distribution (Fig. 4) of locus separation accuracy and ADO number show that the ADO number increased from a gradient of 1:4 and peaked at gradients of 1:7, 1:8 and 1:9. The correlation coefficient of mixed gradient and locus separation accuracy was estimated at R^2^=−0.7121 (P=0.03139), indicating a negative linear correlation between these two parameters. The correlation coefficient of mixed gradient and ADO number was estimated at R^2^=−0.4244 (P=0.2549), demonstrating no significant correlation between these two parameters. Fig. 5 shows the distribution of average locus separation accuracy at different mixed gradients in the three parallel tests, in which the results were generally consistent. Locus separation accuracy was lowest at a mixed gradient of 1:1; with an increasing mixed gradient, the accuracy first increased and then decreased. Specifically, locus separation accuracy was relatively high at gradients of 1:2, 1:3 and 1:4 but decreased to low levels and fluctuated at gradients of 1:1 and 1:9. The accuracy was slightly higher in mixed DNA profiles excluding loci with ADO compared with those including ADO.

**Figure 6:**
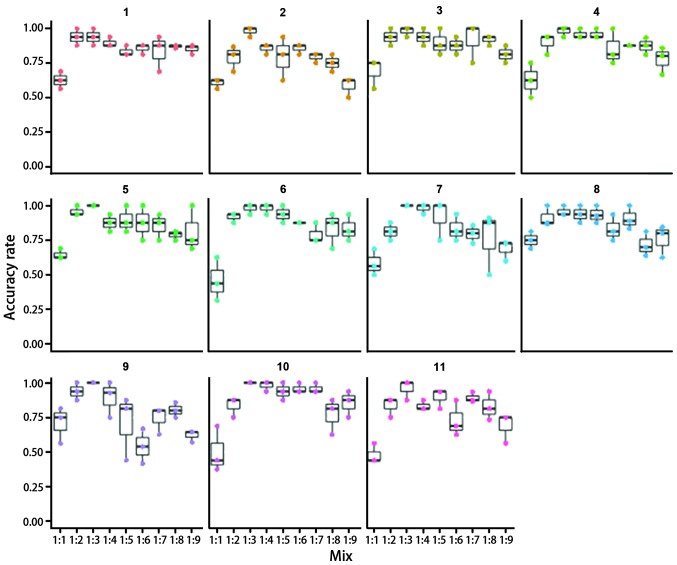
Box-whisker plots of the average locus separation accuracy in eleven groups of male-mixed DNA with nine mixed gradients.

Data statistics (Table VII) and distribution (Fig. 6) of locus separation accuracy in the eleven groups of male-mixed DNA samples at different mixed gradients show that the distribution pattern of the accuracy in every group of mixed DNA was generally consistent with the overall distribution mentioned above. The accuracy was lowest at a gradient of 1:1 (with the exception of no. 9). With an increasing mixed gradient, the accuracy first increased and then decreased. Among the eleven groups of mixed-DNA, large fluctuations in locus separation accuracy were observed in groups no. 7, 9 and 11, which may have been due to variations in experimental operations. The accuracy was generally high in groups no. 1, 3 and 4. There were differences in the overall level of locus separation accuracy among the eleven groups of mixed DNA, demonstrating the stochastic effect of sampling.

#### Vertical analysis

**Figure 7:**
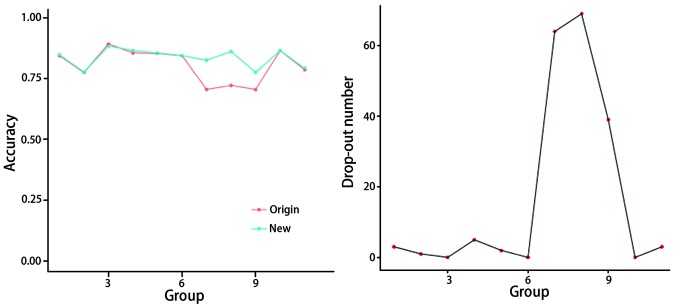
Distribution of locus separation accuracy (left) and allele drop-out number (right) in the eleven groups of male-mixed DNA. The green line represents the distribution pattern excluding STR loci with drop-out and the red line represents the pattern including STR loci with drop-out. STR, short tandem repeats.

Each group of male-mixed DNA profiles (with three parallel tests) involved 432 STR loci. Data statistics (Table VIII) and distribution (Fig. 7) of locus separation accuracy and the ADO number show that the accuracy was generally high (>80%) for the eleven groups of mixed DNA, with the exception of groups no. 2, 9 and 11. Due to low average peak heights of the active alleles (APH), the ADO number of STR loci was significantly greater in groups no. 7, 8 and 9 than it was in the other groups. In addition, there were large differences in locus separation accuracy, including and excluding loci with ADO (~10%).

**Figure 8:**
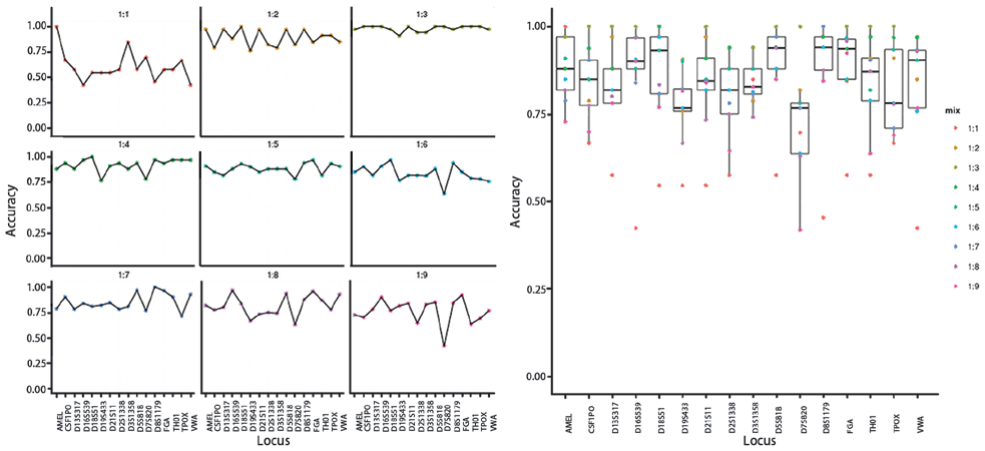
Line charts and box-whisker plots of separation accuracy for 16 short tandem repeats loci in male-mixed DNA profiles at nine mixed gradients.

In the mixed DNA experimental model, nine mixed gradients of a specific locus involved 33 values of locus separation accuracy. Data statistics (Table IX) and distribution (Fig. 8) of locus separation accuracy for 16 STR loci at each mixed gradient show that for a gradient of 1:1, the accuracy was ≤70% for the STR loci, with the exception of AMEL- and D3S1358 (outliers are shown in the lower area of the box-whisker plot, Fig. 8). For gradients of 1:2, 1:3, 1:4 and 1:5, the accuracy of each locus was relatively high, particularly at the gradient of 1:3 (≥90%), while at the gradients of 1:8 and 1:9, the accuracy underwent large fluctuations and declined to lower levels. According to the data distribution shown in the box-whisker plot, the average separation accuracy was lowest for the D7S820 locus among the 16 STR loci.

**Figure 9:**
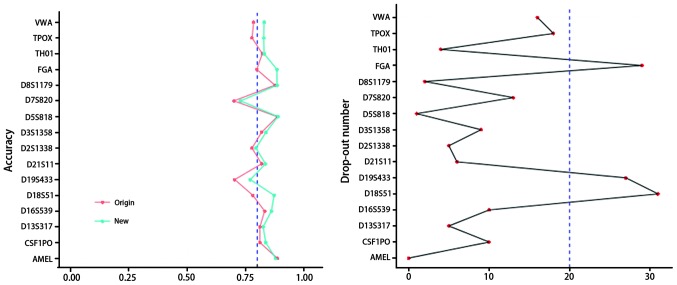
Distribution of overall separation accuracy (left) and allele drop-out number (right) for 16 STR loci in male-mixed DNA profiles. The green line represents the distribution pattern excluding STR loci with drop-out, the red line represents the pattern including STR with drop-out and the blue dotted line indicates an accuracy of 0.8 and drop-out number of 20, respectively. STR, short tandem repeats.

Data statistics (Table X) and distribution (Fig. 9) of locus separation accuracy in the 297 simulated male-mixed DNA profiles show that the accuracy was relatively high for loci D5S818, D8S1179 and FGA (>88%), but relatively low for loci D19S433, D2S1338 and D7S820 (≤80%). The number of ADO was lowest in AMEL-, D5S818 and D8S1179, but was relatively high in loci D18S51, D19S433, FGA, TPOX and vWA (>15). The latter five loci were all distributed in the yellow and red channels with lower APH, consistent with the relatively low sensitivity to yellow and red fluorescence in the ABI 3130xl Genetic Analyzer. There was no significant correlation between the accuracy of the 16 STR loci and number of ADO, R^2^=−0.3095 (P=0.2434).

**Figure 10:**
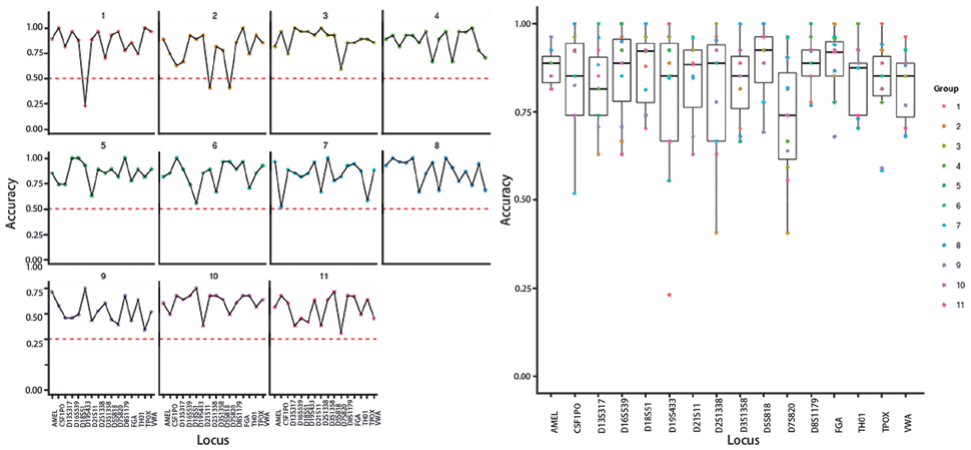
Line charts and box-whisker plots of separation accuracy for 16 short tandem repeats loci in eleven groups of male-mixed DNA profiles. The red line indicates an accuracy of 0.5.

Data statistics (Table XI) and distribution (Fig. 10) of locus separation accuracy in the eleven groups of simulated male-mixed DNA profiles show that groups no. 1, and 7 contained relatively large numbers of loci corresponding to the separation accuracy ≤0.5. The accuracy of loci D19S433, D2S1338 and D7S820 were associated with relatively large fluctuations, with the lowest median accuracy for D7S820. These results were generally consistent with the overall distribution of locus separation accuracy at the nine mixed gradients in the results from the other experiments.

## Discussion

In the present study, an experimental model comprising eleven groups of male-mixed DNA (n=297) was established by following the mixed DNA construction principle of using an ABI 7500 real-time PCR system with scientific validation of the Mx parameter (RMSE≤0.02). The locus separation accuracy of the mixsep package was statistically analyzed using horizontal and vertical analysis of experimental data, with mixed DNA profiles and STR loci as units. The DNA profile distribution data corresponding to different mixed gradients, STR loci and mixed DNA types was examined to assess the performance of the mixsep package in the analysis of mixed DNA.

Locus separation accuracy of mixsep had a negative linear correlation with the Mx value (R^2^=−0.7121, with the exception of the gradient, 1:1, which first increased and then decreased with increasing mixed gradient imbalance. Thus, the Mx value was one of the primary factors that determined the success of mixed DNA analysis. Among the 16 STR loci, the number of ADO was relatively high in the D18S51, D19S433, FGA, TPOX and vWA loci (>15). These five loci were all located in the yellow and red channels and had a low APH, consistent with the low sensitivity to yellow and red fluorescence of the ABI 3130xl Genetic Analyzer. In addition, there was a large non-significant difference in locus separation accuracy obtained depending on whether the loci with ADO were included or excluded (~10%). The presence of ADO reduced the analytical performance of mixsep, consistent with the lack of ADO functional modules in this software.

The present study demonstrated that the mixsep software had a number of advantages. It was easy to operate and produced understandable results with a degree of controllability. It produced intuitive results presented in visual typing maps. Furthermore, rational assumptions were made in the established model with appropriate reasoning, and produced results with high validity. However, certain limitations remained in the use of mixsep, including the existence of bugs, which may result in the occasional generation of outliers in data analysis, as well as graphic dysfunction. In addition the control of software interface was inflexible and presentation was occasionally incomplete. Due to these limitations, the lack of analysis modules for dealing with stutter, drop-out and drop-in, and the unknown prior conditions in model assumptions, it is necessary to further optimize and improve the mixsep package in order to produce consistently reliable results.

## Figures and Tables

**Table I:** DNA concentration in eleven groups of male-mixed DNA.

Sample no.	Sample 1		Sample 2		Difference (ng/*µ*l)
	Person no.	Concentration (ng/*µ*l)	Person no.	Concentration (ng/*µ*l)	
1	1	5.80	3	5.75	0.05
2	11	7.20	40	7.21	0.01
3	11	7.20	14	7.22	0.02
4	20	9.24	26	9.35	0.11
5	12	6.58	19	6.68	0.10
6	24	5.46	27	5.48	0.02
7	4	6.92	15	6.98	0.06
8	7	6.13	29	6.22	0.09
9	11	7.20	38	7.14	0.06
10	37	6.40	39	6.39	0.01
11	9948	0.223	2800M	0.243	0.02

**Table II:** Mixed gradients of simulated male-mixed DNA.

Male-mixed DNA	Mixed gradient								
	1:1	1:2	1:3	1:4	1:5	1:6	1:7	1:8	1:9
Volume sample 1 (*µ*l)	5	4	3	2	2	2	2	2	2
Volume sample 2 (*µ*l)	5	8	9	8	10	12	14	16	18

**Table III:** DNA quantity in eleven groups of male-mixed DNA with mixed gradient of 1:9.

Sample no.	Quantity mixed DNA (ng/*µ*l)	Sample 1		Sample 2		Difference (ng/*µ*l)
		Person no	Concentration (ng/*µ*l)	Person no	Concentration (ng/*µ*l)	
1	8.32	1	5.80	3	5.75	2.57
2	10.94	11	7.20	40	7.21	3.74
3	10.51	11	7.20	14	7.22	3.31
4	13.38	20	9.24	26	9.35	4.14
5	9.60	12	6.58	19	6.68	3.02
6	7.01	24	5.46	27	5.48	1.55
7	9.62	4	6.92	15	6.98	2.70
8	8.68	7	6.13	29	6.22	2.55
9	10.38	11	7.20	38	7.14	3.24
10	9.31	37	6.40	39	6.39	2.92
11	0.337	9948	0.223	2800M	0.243	0.114

The difference is the quantity of mixed DNA minus the concentration from either sample 1 or sample 2, depending on which value was smallest.

**Table IV:** Dilutions of male-mixed DNA working solutions^a^.

Sample no.	DNA quantity (ng/*µ*l)	Dilution factor	PCR template (*µ*l)
1	8.32	10x	1
2	10.94	10x	1
3	10.51	10x	1
4	13.38	15x	1
5	9.60	10x	1
6	7.01	10x	1
7	9.62	10x	1
8	8.68	10x	1
9	10.38	10x	1
10	9.31	10x	1
11	0.337	1x	2

a Target concentration of DNA template, 0.5–1.25 ng/*µ*l. PCR, polymerase chain reaction.

**Table V:** RMSE of estimated alpha in eleven groups of male-mixed DNA profiles with different mixed gradients.

Gradient	1:1	1:2	1:3	1:4	1:5	1:6	1:7	1:8	1:9
Theoretical alpha	0.5000	0.3333	0.2500	0.2000	0.1667	0.1429	0.1250	0.1111	0.1000
RMSE of est. alpha									
1	0.0124	0.0062	0.0117	0.0152	0.0083	0.0120	0.0066	0.0101	0.0019
2	0.0044	0.0153	0.0112	0.0076	0.0215	0.0064	0.0073	0.0096	0.0124
3	0.0048	0.0251	0.0051	0.0051	0.0097	0.0088	0.0118	0.0100	0.0076
4	0.0030	0.0310	0.0135	0.0082	0.0036	0.0094	0.0085	0.0135	0.0131
5	0.0066	0.0148	0.0104	0.0181	0.0074	0.0109	0.0053	0.0061	0.0140
6	0.0116	0.0027	0.0030	0.0039	0.0068	0.0051	0.0099	0.0196	0.0132
7	0.0045	0.0249	0.0065	0.0027	0.0072	0.0157	0.0082	0.0147	0.0059
8	0.0108	0.0121	0.0133	0.0110	0.0225	0.0187	0.0020	0.0117	0.0052
9	0.0491	0.0214	0.0027	0.0156	0.0053	0.0122	0.0037	0.0248	0.0072
10	0.0127	0.0140	0.0143	0.0125	0.0085	0.0089	0.0096	0.0109	0.0103
11	0.0054	0.0391	0.0106	0.0101	0.0074	0.0073	0.0203	0.0150	0.0117
Total	0.0267	0.0130	0.0160	0.0179	0.0148	0.0126	0.0175	0.0177	0.0182

RMSE, root mean square error; est., estimated.

**Table VI:** Statistics of locus separation accuracy and ADO number in male-mixed DNA at different mixed gradients.

Mix	1:1	1:2	1:3	1:4	1:5	1:6	1:7	1:8	1:9
Accuracy	0.6061	0.8878	0.9791	0.9181	0.8809	0.8304	0.8510	0.8166	0.7557
Drop no	0	2	2	15	16	9	38	59	45
Loci no.	528	526	526	513	512	519	490	469	483
Sum	528	528	528	528	528	528	528	528	528

ADO, allelic drop-out.

**Table VII:** Statistics of locus separation accuracy in eleven groups of male-mixed DNA samples at different mixed gradients.

Mix	Group										
	1	2	3	4	5	6	7	8	9	10	11
1:1	0.6250	0.6042	0.6875	0.6250	0.6458	0.4583	0.5833	0.7500	0.7083	0.5000	0.4792
1:2	0.9375	0.7872	0.9375	0.8958	0.9583	0.9167	0.8125	0.9149	0.9375	0.8333	0.8333
1:3	0.9375	0.9792	0.9792	0.9792	1.0000	0.9792	1.0000	0.9574	1.0000	1.0000	0.9583
1:4	0.8958	0.8542	0.9375	0.9583	0.8750	0.9792	0.9767	0.9250	0.8913	0.9792	0.8333
1:5	0.8333	0.7917	0.8958	0.9583	0.8958	0.9375	0.9189	0.9302	0.7083	0.9375	0.8958
1:6	0.8542	0.8542	0.8750	0.8542	0.8750	0.8750	0.8333	0.8298	0.5500	0.9583	0.7292
1:7	0.8333	0.7917	0.9167	0.8750	0.8511	0.7917	0.8000	0.9286	0.7391	0.9583	0.8913
1:8	0.8696	0.7500	0.9167	0.8723	0.7872	0.8333	0.8000	0.7297	0.7857	0.7708	0.8298
1:9	0.8511	0.5833	0.8125	0.7727	0.8125	0.8333	0.6944	0.7692	0.6190	0.8542	0.6875

**Table VIII:** Statistics of overall locus separation accuracy and ADO number in eleven groups of male-mixed DNA.

Group	1	2	3	4	5	6	7	8	9	10	11
Accuracy	0.8485	0.7773	0.8843	0.8665	0.8558	0.8449	0.8261	0.8623	0.7761	0.8657	0.7925
Drop no.	3	1	0	5	2	0	64	69	39	0	3
Loci no.	429	431	432	427	430	432.	368	363	393	432	429
Sum	432	432	432	432	432	432	432	432	432	432	432

ADO, allelic drop-out.

**Table IX:** Statistics of separation accuracy for 16 STR loci in male-mixed DNA profiles with different mixed gradients.

Mix	AMEL-	CSF1PO	D13S317	D16S539	D18S51	D19S433	D21S11	D2S1338	D3S1358	D5S818	D7S820	D8S1179	FGA	TH01	TPOX	vWA
1:1	1.0000	0.6667	0.5758	0.4242	0.5455	0.5455	0.5455	0.5758	0.8485	0.5758	0.6970	0.4545	0.5758	0.5758	0.6667	0.4242
1:2	0.9697	0.7879	0.9697	0.8788	1.0000	0.7576	0.9697	0.8182	0.7879	0.9697	0.8182	0.9697	0.8438	0.9091	0.9091	0.8485
1:3	0.9697	1.0000	1.0000	1.0000	0.9697	0.9062	1.0000	0.9394	0.9394	1.0000	1.0000	0.9697	1.0000	1.0000	1.0000	0.9697
1:4	0.8788	0.9375	0.8788	0.9688	1.0000	0.7667	0.9091	0.9375	0.8788	0.9394	0.7812	0.9697	0.9355	0.9697	0.9677	0.9677
1:5	0.9091	0.8485	0.8182	0.8788	0.9310	0.9000	0.8485	0.8788	0.8788	0.8788	0.7812	0.9394	0.9667	0.8182	0.9333	0.9032
1:6	0.8485	0.9032	0.8182	0.9062	0.9688	0.7667	0.8182	0.8182	0.8125	0.8788	0.6364	0.9394	0.8485	0.7879	0.7812	0.7576
1:7	0.7879	0.9032	0.7812	0.8387	0.8077	0.8214	0.8438	0.7812	0.8065	0.9697	0.7667	1.0000	0.9630	0.9032	0.7097	0.9310
1:8	0.8182	0.7742	0.8000	0.9667	0.8333	0.6667	0.7333	0.7500	0.7419	0.9375	0.6296	0.8750	0.9583	0.8710	0.7778	0.9286
1:9	0.7273	0.7000	0.7812	0.9000	0.7692	0.8148	0.8387	0.6452	0.8276	0.8485	0.4194	0.8438	0.9231	0.6364	0.6897	0.7667

STR, short tandem repeats.

**Table X:** Overall separation accuracy and ADO number of 16 STR loci in male-mixed DNA profiles.

Locus	AMEL-	CSF1PO	D13S317	D16S539	D18S51	D19S433	D21S11	D2S1338	D3S1358	D5S818	D7S820	D8S1179	FGA	TH01	TPOX	vWA
Accuracy	0.8788	0.8362	0.8253	0.8606	0.8722	0.7704	0.8351	0.7945	0.8368	0.8885	0.7289	0.8847	0.8843	0.8294	0.8280	0.8292
Drop no.	0	10	5	10	31	27	6	5	9	1	13	2	29	4	18	16
Loci no.	297	287	292	287	266	270	291	292	288	296	284	295	268	293	279	281
Sum no.	297	297	297	297	297	297	297	297	297	297	297	297	297	297	297	297

ADO, allelic drop-out; STR, short tandem repeats.

**Table XI:** Statistics of separation accuracy for 16 STR loci in 11 groups of male-mixed DNA profiles.

Group	AMEL-	CSF1PO	D13S317	D16S539	D18S51	D19S433	D21S11	D2S1338	D3S1358	D5S818	D7S820	D8S1179	FGA	TH01	TPOX	vWA
1	0.8889	1.0000	0.8148	0.9630	0.8800	0.2308	0.8889	0.9630	0.7037	0.9259	0.9630	0.7778	0.8519	0.7407	1.0000	0.9630
2	0.8889	0.7407	0.6296	0.6667	0.9231	0.8889	0.9259	0.4074	0.8148	0.7778	0.4074	0.8519	1.0000	0.7407	0.9259	0.8519
3	0.8148	0.9630	0.7407	1.0000	0.9630	0.9630	0.9259	1.0000	0.9259	0.9259	0.5926	0.8519	0.8519	0.8889	0.8889	0.8519
4	0.8889	0.9231	0.8148	0.9259	0.9231	0.8519	0.9630	0.6667	0.8889	0.9630	0.6667	0.963	0.9583	1.0000	0.7778	0.7037
5	0.8519	0.7407	0.7407	1.0000	1.0000	0.9259	0.6296	0.8889	0.8519	0.8889	0.8148	1.0000	0.7778	0.8889	0.8148	0.8889
6	0.8148	0.8519	1.0000	0.8889	0.7407	0.5556	0.8519	0.8889	0.6667	0.9630	0.9630	0.8889	0.9630	0.7037	0.8519	0.9259
7	0.9630	0.5185	0.8846	0.8519	0.8125	0.8462	0.9600	0.6667	1.0000	0.7778	0.8182	0.9231	0.9412	0.8750	0.5833	0.8824
8	0.9259	1.0000	0.9615	0.9500	1.0000	0.6667	0.8462	0.9545	0.6818	1.0000	0.9048	0.7692	0.8667	0.7308	0.9412	0.6818
9	0.9630	0.8261	0.7083	0.7083	0.7391	1.0000	0.6800	0.7778	0.8519	0.6923	0.6400	0.9259	0.6800	0.8889	0.5909	0.7692
10	0.8519	0.7407	0.9259	0.8889	0.9259	1.0000	0.6296	0.9259	0.9259	0.8889	0.7407	0.8519	0.9259	0.9259	0.8148	0.8889
11	0.8148	0.9259	0.8519	0.6296	0.7037	0.6667	0.8846	0.6296	0.8889	0.9630	0.5556	0.9259	0.9200	0.7407	0.8889	0.7037

STR, short tandem repeats.

## References

[1] R Development Core Team (2013) (2013). R: A language and environment for statistical computing.

[2] RStudio (2012) (2012). RStudio: Integrated development environment for R (Version 0.96.122).

[3] Gentleman RC, Carey VJ, Bates DM (2004). Bioconductor: open software development for computational biology and bioinformatics. Genome Biol.

[4] Smyth GK (2005). Limma: linear models for microarray data. Bioinformatics and computational biology solutions using R and Bioconductor.

[5] Gautier L, Cope L, Bolstad BM, Irizarry RA (2004). affy - analysis of Affymetrix GeneChip data at the probe level. Bioinformatics.

[6] Ellis B, Haaland P, Hahne F, Le Meur N (2009). basic structures for flow cytometry data. R package version 1.24.2.

[7] Pages H, Carlson M, Falcon S, Li N, Maintainer MBP (2014). Package ‘AnnotationDbi’: Annotation Database Interface. R package version 1.20.7.

[8] Carlson M (2012). hgu95av2.db: Affymetrix human genome U95 set annotation data (chip hgu95av2). R package version 2.8.0.

[9] Carlson M, Falcon S, Pages H (2010). A set of annotation maps describing the entire Gene Ontology. R package version 2.8.0.

[10] Shannon P (2012). MotifDb: An annotated collection of protein-DNA binding sequence motifs. R package version 1.0.0.

[11] Pages H, Aboyoun P, Gentleman R, DebRoy S (2009). String objects representing biological sequences, and matching algorithms. R package version 2.26.3.

[12] Tvedebrink T (2013). mixsep: DNA mixture separation. R package version 0.2.1-2.

[13] Tvedebrink T (2011). mixsep: An R-package for DNA mixture separation. Forensic Sci Int: Genet Supple Seri.

[14] Tvedebrink T, Eriksen PS, Mogensen HS, Morling N (2012). Identifying contributors of DNA mixtures by means of quantitative information of STR typing. J Comput Biol.

[15] Tvedebrink T, Eriksen PS, Mogensen HS, Morling N (2010). Evaluating the weight of evidence by using quantitative short tandem repeat data in DNA mixtures. J R Stat Soc Ser C Appl Stat.

